# How to Regulate the Right to Self-Medicate

**DOI:** 10.1007/s10730-020-09415-7

**Published:** 2020-06-03

**Authors:** Joseph T. F. Roberts

**Affiliations:** University of Birmingham, Birmingham, UK

**Keywords:** Competence, Harm reduction, Pharmaceutical, Regulation, Self-medication

## Abstract

In *Pharmaceutical Freedom* Professor Flanigan argues we ought to grant people self-medication rights for the same reasons we respect people’s right to give (or refuse to give) informed consent to treatment. Despite being the most comprehensive argument in favour of self-medication written to date, Flanigan’s *Pharmaceutical Freedom* leaves a number of questions unanswered, making it unclear how the safe-guards Flanigan incorporates to protect people from harming themselves would work in practice. In this paper, I extend Professor Flanigan’s account by discussing a hypothetical case to illustrate how these safe-guards could work together to protect people from harms caused by their own ignorance or incompetence.

## Introduction

In *Pharmaceutical Freedom* Professor Flanigan presents the most comprehensive argument for why prohibitionist approaches to drug regulation are wrong written to date. The crux of Flanigan’s argument is that policies prohibiting people from accessing certain drugs are incompatible with the doctrine of informed consent. If people have a right to refuse medical treatment (even when doing so could damage their health), they should also have a right to self-medicate (even when this means they put their health at risk).

Flanigan argues respecting people’s right to self-medicate requires: (i) ending prohibitionist approaches to the regulation of recreational drugs, (ii) abolishing mandatory prescription requirements, and (iii) abolishing pre-market testing requirements. In Flanigan’s proposal, institutions such as the Federal Drugs Administration (FDA) and the European Medicines Agency (EMA) would act as certification bodies. Their role would be to test drugs and issue recommendations based on the results of their testing. The main difference between this and the current system is that these bodies would no longer be entitled to force consumers to comply with their recommendations.

Despite being the best defence of the right to self-medication to date, Flanigan’s *Pharmaceutical Freedom* leaves a number of practical questions unanswered. For example: how should information be made accessible to consumers? How should consumer’s competence be assessed for behind the counter drugs?

In this paper, I aim to extend Flanigan’s framework by outlining how people’s rights to self-medication could work in practice, drawing out the implications of her view. The reason this is important is that getting clear on how rights of self-medication should be implemented at a policy level is a crucial step in the process of testing the plausibility of the argument. Unless we can lay to rest doubts about liberalised access to drugs leading to people unknowingly or incompetently harming themselves, the case for granting people rights to self-medication will be weakened. In order for the changes advocated in Flanigan's book to become a reality in a democratic society, they will need extensive public support. To generate this support, we need a hypothetical view of what the world would look like without prescription requirements, pre-market testing requirements and the almost complete prohibition of recreational drugs.

My goal in this piece is to provide such a view by illustrating what interactions between pharmacists and consumers could look like under a certification system like Flanigan’s. The aim is to provide a view of how a hypothetical liberalised market for drugs could work and to illustrate the safeguards and harm-reduction policies that are compatible with respecting people’s self-medication rights. The hope is that by illustrating how these safeguards could work together, we will have a better idea of how they might protect people from unwanted harms without interfering with their rights to self-medicate.

The structure of the paper is as follows. In "Pharmaceutical Freedom", I re-construct Professor Flanigan’s theoretical arguments and bring together the policy recommendations contained within *Pharmaceutical Freedom*. In "Some Unanswered Questions", I expand on why more detailed policy proposals are needed by identifying a series of questions which *Pharmaceutical Freedom* leaves unanswered. In "Robertsville", I discuss a series of hypothetical cases to illustrate how these regulations could work in practice, the aim being to show that policies aimed at protecting people from harming themselves are compatible with liberalised access to pharmaceuticals. Finally, I conclude by arguing that, if suitably extended, Flanigan’s proposal for drug liberalisation could strike a plausible balance between people’s self-medication rights and society’s duty to protect people from harms arising from their own ignorance or incompetence. Whether it does so in practice, I suggest, will depend on the enforcement regime policymakers choose to adopt to ensure compliance.

## Pharmaceutical Freedom

The crux of Professor Flanigan’s argument in *Pharmaceutical Freedom* is that the way in which we regulate people’s access to drugs (i.e., through prohibitions, prescription requirements, and a regime of pre-market testing) is at odds with the doctrine of informed consent. To illustrate, Flanagan asks us to consider a case involving two people making decisions about medical treatment for diabetes:

Diabetes: Frida and Karl both have diabetes and are both visiting their respective doctors. Frida’s doctor tells her to treat it with insulin. Frida dislikes this option because it will force her to live by a restrictive schedule and says she would prefer to manage her diabetes through diet and exercise alone. Although Frida’s physician may consider that she is putting her health at risk, Frida has both a legal and moral right to refuse treatment. If her physician deceived her into taking insulin, they would commit a grave wrong, for they would be acting without their patient’s informed consent. Things go differently for Karl. When he visits his physician for a consultation on how to manage diabetes, his physician recommends that he do so through diet and exercise alone. Not being an active man, Karl says he will find exercise difficult and probably won’t keep it up. Instead, he would rather get insulin treatment. His physician objects to providing this course of treatment on the basis that this is beyond the scope of what his professional association considers to be legitimate medical care. Given that his licence can be revoked by them for not complying with their standards of care, the physician informs Karl that he is duty bound to refuse to write him a prescription. As a consequence, Karl is precluded from accessing his preferred treatment as no pharmacist will dispense it to him without a valid prescription.

Flanigan argues that these two cases are morally analogous as “a person does not forfeit their moral status and authority to consent when she steps outside the clinic and into the pharmacy” (Flanigan 2017, p. 31). As a consequence the same moral reasons which support allowing Frida to choose a regime of diet and exercise over insulin treatment support allowing Karl to do the reverse. This puts people who support the current state of affairs in a compromising position. Given that the doctrine of informed consent and self-medication rights stand or fall together, denying that Karl should be entitled to access insulin requires giving up on the doctrine of informed consent; a principle at the heart of liberal bioethics (May 2002, p. 9, 2005, p. 302).

Although giving up on the doctrine of informed consent is a possible response to Flanigan’s incoherence argument, it is too high a price to pay. The doctrine of informed consent is simply too well supported by too wide a variety of reasons. In defence of this claim, Flanigan outlines both an epistemological-consequentialist argument and a series of deontological considerations in favour of granting people rights to make decisions about their own bodies.

The consequentialist case in favour of both the doctrine of informed consent and self-medication rights is that they both enable people to choose what is in their overall best interest (Flanigan 2017, p. 5). Prohibiting people from accessing drugs, on the other hand, forces people to live their lives in accordance with the views of physicians, pharmacists, and regulators. While physicians and pharmacists are experts in health, medical care, and the effects of (certain) drugs, they do not necessarily have access to the all of the information needed to determine whether a treatment advances a particular patient’s best interests (Flanigan 2017, p. 11).

Making treatment choices requires weighing up information about side effects and how these will affect one’s life. What constitutes the right decision for a particular patient will depend on how they weigh their interest in health against the other interests they may have. After all, for most of us health is only one value among many (Flanigan 2017, p. 7) and how to weigh it against others (like recreation or sporting prowess) is a decision each person must make for themselves. For most people the goal is not maximal health for maximal time.

As a consequence, regulators are “not well placed to judge whether using an investigational drug is acceptably safe for a large and heterogeneous patient population whose values and circumstances differ” (Flanigan 2017, p. 27). Although individual physicians may know their patients’ life plans and circumstances better than regulators (especially if they have been in their care for a long time), they too lack the requisite knowledge to judge the right medical decision to make. Requiring a prescription to access certain drugs serves to “privilege physicians’ and pharmacists’ judgements about treatments instead of patients’ judgements about whether using a drug is the right choice” (Flanigan 2017, p. 28). Given that patients themselves are generally better placed to make the decision than physicians, pharmacists, and regulators; protecting people’s rights to make their own medical decisions will likely lead to more wellbeing than granting authority over medical decisions to people’s physicians.

In addition to this epistemic-consequentialist argument, Flanigan provides a deontological defence of both the doctrine of informed consent and self-medication rights. In *Diabetes,* both Karl and Frida are making intimate decisions about their bodies. Given the central role our bodies play in how we live our lives, our self-conception and what we are able to do, there are good reasons to consider choices about our body especially significant. As Flanigan puts it: “Medical choices are bodily, ailments and disease are often impossible to ignore, and a person’s health has a substantial impact on every other choice and plan she makes" (Flanigan 2017, p. 15). In short, we are embodied agents and, as a consequence, what happens to our bodies affects both what we do with our lives and how well they go. Responding adequately to people’s essential embodied nature requires granting them control over their bodies so that they can make decisions in light of their values.

The doctrine of informed consent and rights to self-medication serve to protect one of the core principles of liberalism and, by extension, liberal bioethics: the idea that (in general) people should be allowed to live their own lives in accordance with their own values (Beauchamp 2017; May 2002, p. 2, 2005, p. 302; Taylor 2005, p. 20). Although there are limitations on these rights, such as when doing so harms others (Flanigan 2017, p. 88), there is an overlapping philosophical consensus on the idea that in liberal societies people should not be interfered with unless there is a compelling reason for doing so. For the most part, liberals allow others to do as they please (May 2002, p. 5; Noggle 1997, p. 509).

Of the two types of justification, Flanigan considers the deontological one the strongest; the reason being the epistemological-consequentialist argument is empirically contingent in a way that the deontological one isn’t. The epistemological argument relies on the premise that people are in general able to make their own medical decisions by using the evidence they are given to make decisions under conditions of uncertainty. Now, we all suffer from cognitive biases, use mental short-cuts, and assess risk inaccurately; the crux of the argument is the claim that regulators, pharmacists and physicians are in an even worse epistemic position. If this were reversed (and medical professionals became reliably better at satisfying people’s interests than people themselves), the epistemological-consequentialist argument would support paternalistic interference (Conly 2012). As the deontological defence isn’t vulnerable to these problems, it provides a sturdier foundation on which to ground the doctrine of informed consent and self-medication rights.

However, the deontological argument’s invulnerability to empirical evidence shouldn’t be overstated. If granting everyone rights to self-medication were a reliable way of reducing their wellbeing, or it led to catastrophic consequences for substantial numbers of people, the deontological argument would become increasingly implausible. The case for self-medication rights is at it’s strongest when there is an overlapping consensus between the epistemological-consequentialist argument and the deontological one. The challenge facing any proposal for drug liberalisation is ensuring that both arguments still pull in broadly the same direction; balancing people’s right to self-medicate against society’s general duties to prevent people from harming themselves through ignorance or as a result of their lack of decision-making competence.

In *Pharmaceutical Freedom,* Flanigan accepts that society has a duty to prevent people from unknowingly harming themselves, but argues that prohibitive drugs policies are not required to achieve this goal. Instead Flanigan argues we should “forbid manufacturers from knowingly withholding information about the risks of a drug or from misleading patients about the risk of a treatment” (Flanigan 2017, p. 26). If manufacturers disclose the risks of their drugs, the argument goes, people will be able to avoid dangerous drugs (even if they are available for purchase), in much the same way people avoid ingesting bleach.

This duty to disclose, however, is on shaky grounds. Flanigan argues that manufacturers have an obligation to disclose information because patients have rights not to be deceived about what they are purchasing. As a consequence, the duty to disclose only applies when the norm is that manufacturers disclose all relevant information about the risks of using their products. According to Flanigan, if manufacturers issue a disclaimer that they knowingly withhold information about risks (or if such a situation becomes the norm), failure to disclose is no longer deceptive. So long as individuals are aware that they are consenting to unknown risks, their consent is not invalidated by their ignorance, and manufacturers would not have a duty to disclose the risks of the drugs they manufacture.

To bolster these minimalist disclosure requirements, Flanigan argues that drug certification bodies could promote consumer safety by certifying drugs, implementing information campaigns aimed at discouraging consumers from purchasing uncertified medications, and enforcing labelling and disclosure requirements. To ensure pharmaceutical companies comply with the regulations, they could be made liable for risks they knew about but failed to disclose should people come to harm (Flanigan 2017, p. 36).

To prevent people who are incompetent from harming themselves, Flanigan supports designating dangerous drugs as "behind the counter so that pharmacists can screen for capacity when they sell drugs” (Flanigan 2017, p. 43). Individuals who fail these capacity assessments would then be denied access to the drugs they wish to purchase until they could demonstrate they were competent. Flanigan argues that unlike prohibitionist policies which apply to all people, prohibiting incompetent people from making their own choices about potentially harmful drugs is permissible in virtue of their lack of decision-making competence. Instead, designated guardians ought to determine what is in the incompetent person’s best interest and choose for them.

“Such an approach”, Flanigan writes, “would make all the gatekeeping functions of the prescription drug system available to patients who would prefer not to have legal and easy access to opioids, while allowing other voluntary drug users to purchase and use recreational drugs, a right that is protected by the more general right of self-medication” (Flanigan 2017, p. 81).

To summarise, Flanigan argues that respecting competent adult’s self-medication rights requires the abolition of pre-market testing requirements, prescription requirements, and the legalisation of drugs which are currently prohibited, some of which are dangerous and some of which are relatively innocuous. To help consumers navigate this liberalised marketplace for drugs and make informed choices, Flanigan proposes keeping agencies such as the FDA or the EMA, but changing their role from regulators to certification bodies. Their task would be to test drugs, publish the results, inform consumers about the dangers of untested drugs, and enforce labelling requirements. Individuals who wanted further expert advice would also be free (but not required) to consult a physician and have them recommend a course of treatment. To ensure that children and incompetent adults can’t access dangerous drugs without their guardian’s consent, these would be placed behind the counter, thereby giving pharmacists an opportunity to screen for competence.

In conclusion, Flanigan doesn’t advocate a completely laissez-faire approach to the regulation of drugs. The proposal is more nuanced and includes measures such as mandatory competence assessments and labelling requirements aimed at protecting vulnerable people from potential sources of harm. The question many opponents to drug liberalisation will have is: are these measures enough to protect people who wish to remain safe from harming themselves, whether unknowingly or as a result of their incompetence?

My aim in this paper isn’t to challenge the fundamental arguments in favour of liberalised access to drugs or argue against the broad outline of the certification system Flanigan proposes. I am sympathetic to both and, like Flanigan, believe that public officials in liberal societies are required to respect people’s decisions about their own bodies and that, as a consequence, people should be entitled to access the drugs they wish to consume (even when they are deadly, dangerous, and/or untested). My goal is the much more modest one of sympathetically extending the framework and illustrating how it could work in practice in the hopes of allaying concerns about liberalisation. The first step in doing so is identifying where Flanigan’s account would benefit from being extended. This is the task of section "Some Unanswered Questions".

## Some Unanswered Questions

Despite being the most exhaustive defence of self-medication rights written to date, some of the policy proposals in *Pharmaceutical Freedom* could benefit from being extended. The reason is that, as it stands, it is difficult to see exactly how the safeguards Flanigan includes to prevent people from harming themselves as a result of ignorance or incompetence are up to the task. In order to see whether the view proposed is plausible, what is needed is a picture of how self-medication rights could work in practice. To do this, we need more detail.

### Ignorance

Let us begin with the problem of people unknowingly harming themselves. Flanigan’s solution to this problem relies on making manufacturers liable for harms arising from risks they do not disclose, certification bodies testing and certifying drugs on the market, and enforcing labelling requirements. When little is known about drugs (e.g., non-tested drugs), manufacturers should make the fact that we lack good information explicit. The aim of these policies is to ensure that people can give morally transformative consent to the person dispensing the drugs. Whatever decision the person takes after having been informed of the risks (or of the fact we don’t know the risks), will have been taken knowingly.

Although forcing manufacturers to make information available may help some people become more informed about what drugs they are consuming, it is unclear whether this alone is enough to prevent people from unknowingly harming themselves. The reason being that ensuring that information is available in general doesn’t necessarily mean it is accessible to people when they need it in a format they understand. As a consequence, forcing manufacturers to disclose information and funding certification bodies may not be enough to ensure that people are giving their *informed* consent to drug taking. To illustrate, it will be helpful to consider a version of Mill’s (2013) bridge example:

Bridge: Clive is trekking in the forest and comes across a bridge and, assuming it is safe, decides to cross it to see what is on the other side. Unbeknownst to Clive, this particular bridge has been deemed unsafe by the World Bridge Certification Board (which maintains a list of all dangerous bridges on their website and funds "Bridge-danger Awareness" campaigns) and by the bridge owner (as part of their publicly accessible accounts hosted on their website). However, Clive has never visited these websites and, as a consequence, he remains ignorant of the dangers of crossing this particular bridge (even if other people do know it is dangerous). Unaware of the danger, Clive makes his first steps towards the bridge.

The question is: would it be permissible for a more knowledgeable bystander (such as an employee of the World Bridge Certification Board) to stop Clive and inform him of the dangers of crossing the bridge? Flanigan argues that emergency paternalism is warranted in cases like *Bridge* as Clive’s ignorance renders him "a temporarily incompetent bridge crosser" (Flanigan 2017, p. 38). It seems, therefore, that the mere availability of information isn’t sufficient for us to consider Clive informed or voluntarily acting under conditions of ignorance.

Accepting that information needs to be more than simply available raises a series of questions about how information should be conveyed to patients to ensure they are knowingly assuming the risks of using pharmaceuticals. For example: how accessible does information need to be for us to consider a person's consent *informed?* Do suppliers of drugs (for example physicians or pharmacists) have a duty to disclose information about them to people before they dispense them? If they do, can they force people wishing to consume the drugs they supply to sit through the disclosure?

Importantly, the answers to these questions will affect the plausibility of the regulatory system being proposed. If information doesn’t have to be made accessible to people in a format they can understand, requiring that manufacturers disclose risks won’t help all people who wish to avoid dangerous drugs (or bridges) to do so. Similarly, if people can refuse to have information disclosed to them, people who aren’t aware of how little they know about the risks of a drug may harm themselves unknowingly.

Under a certification scheme, people who wish to consume dangerous drugs needn’t acquire a prescription from a physician before purchasing drugs at the pharmacy. Flanigan argues that one of the advantages of this is that it would reduce the cost of medical care, as people who know what is wrong with them and how to treat it would no longer need to engage in the "rubber-stamping" exercise of going to the physician to get a prescription.

However, doing away with mandatory visits to the physician will also likely lead to more people self-diagnosing. Given the complexity of the causal mechanisms involved in choosing drug treatments, they may end up misdiagnosing themselves and requesting medications aimed at treating conditions they do not have. Furthermore, even if they are right about the diagnosis, they may end up choosing ineffective means to their ends, wasting resources purchasing medications they do not need. Moreover, given that many pharmaceuticals have unpleasant side-effects, using the wrong medications to treat a condition can lead to people hindering the pursuit of their goals by exposing themselves to unnecessary harms. Although, in general, people have both the knowledge and the incentive to promote their best interests, this isn’t always the case. This is especially true when the causal connections are complex and difficult to untangle (as they often are in self-medication cases).

In order to help prevent people from harming themselves under a certification scheme, the role of pharmacists in the overall delivery of healthcare will have to change.^1^ Whereas under a regime requiring prescriptions, people would have visited a physician to receive a diagnosis and discuss treatment options before turning up at the pharmacy, under a certification scheme not everyone will, meaning that they may not have had information about the risks of the drugs they are requesting disclosed to them. Although pharmacists already offer advice on how to treat common and non-serious ailments using medications on general sale (such as aspirin or ibuprofen), with the abolition of the prescription requirement they are likely to encounter more people requesting specific drugs on the basis of self-diagnosis, meaning they will be increasingly find themselves being called upon by consumers to provide expert advice to guide them in their self-medication.

Ensuring that people who take risks do so knowingly requires making information about the risks of drugs accessible to the person making the choice, at the time they are making it, in a format they can understand. Under a certification scheme, pharmacists would increasingly find themselves being called upon to perform this task. Although they would have clinical trial data collected by drug certification bodies (such as the reformed FDA Flanigan proposes) to help them inform patients, this goes beyond the role pharmacists currently play in healthcare systems which require prescriptions. Changing the role of pharmacists in this way is therefore likely to require expanding the training of pharmacists to ensure they know how to assess clinical evidence, disclose it to the public, and conduct competence assessments.

This requirement mirrors standard informed consent requirements in healthcare situations, where each individual patient needs to be informed of the risks of the procedure before their consent to treatment is considered morally transformative (Faden, Beauchamp, and King 1986; Manson and O’Neill 2007). Given that under a certification scheme pharmacists might be the only healthcare professional people consult, making information available to consumers will mean that pharmacists need to engage in mandatory information disclosure at the time people purchase pharmaceuticals.

This, however, is still not enough to ensure people don’t unknowingly harm themselves. When people purchase medications at a pharmacy, they normally do not consume them on the premises. Instead, people take them home and, as a consequence, end up consuming them without direct supervision by a healthcare professional. In many cases, people’s ailments require continued pharmacological treatment over a period of time. It might only be days, weeks or months; but it could be years or even a life-time. As these time periods get longer, it gets increasingly easy to forget what the pharmacist said at the time of purchase, making mistakes in self-medication more likely. To reduce the risk of people unknowingly harming themselves by self-medicating, information about the risks of drugs and common ways in which people, unwittingly or otherwise, misuse them should be made publicly available and easily accessible at a later date. Possible ways of doing this include funding hotlines staffed by pharmacists to resolve doubts about how to self-medicate or issuing people with reminders to book appointments with their pharmacists for a medication review.

Now that we’ve seen how Flanigan’s account needs extending to ensure people don’t unknowingly harm themselves, it is time to turn to unanswered questions surrounding the second main safeguard in Flanigan certification scheme: placing drugs behind the counter.

### Incompetence

On Flanigan’s account, placing drugs behind the counter provides an opportunity for pharmacists to screen for capacity, thereby preventing children and incompetent adults from accessing dangerous drugs. What Flanigan’s account doesn’t tell us, however, is exactly how behind the counter dispensing would work in practice. The first thing we need to know is how competence should be assessed. This is important because competence assessments distribute decision-making authority (Brock 1991; Baumgarten 1980; Freedman 1981; Richardson 2010; Skene 1991; Welie 2001). In other words, a judgement that one is competent “commonly functions to denote persons whose consents, refusals and statements of preference will be accepted as binding” (Faden et al. 1986, p. 290).

Given that being found competent determines whether or not someone has rights to self-medication (and consequently whether they can access drugs legally), establishing how competence should be assessed is crucial. Setting the threshold of competence too high means that many of us will be denied the freedom to self-medicate. Setting it too low means individuals who lack decision-making competence will not get the support and protection they deserve.

According to most standard views of competence, whether someone is competent is a matter of whether they possess the capacities to (i) acquire knowledge about the world, (ii) reason instrumentally, and (iii) form and revise a life-plan or conception of the good (Wicclair 1991; Buchanan and Brock 1986; Moye et al 2006; Roberts 2018).^2^ For ease of exposition, let us call these three capacities the Core Capacities (CC). Individuals who can demonstrate that they possess the CCs to the extent necessary to meet a predetermined threshold during the process of decision-making should be entitled to exercise their self-medication rights free from interference.

The first step in ascertaining whether or not someone is competent is establishing the threshold of the Core Capacities the individual must meet to demonstrate competence. On risk-sensitive accounts of competence (Buchanan and Brock 1986; Brock 1991; Drane 1985; Roberts 2018; Skene 1991), the height of the competence threshold is determined by the riskiness of the decision (where risk is determined in light of the values of the person undergoing the assessment). Establishing the height of the threshold requires ascertaining what the person’s values are and how the proposed intervention interacts with these. Given that people’s values can’t be read off their faces, this will require a two-way conversation between the competence assessor (CA) and the person who’s competence is being assessed (PA).^3^

Once the height of the threshold has been established, the second step in conducting a competence assessment is establishing whether the person being assessed (PA) possesses the Core Capacities (CCs) to the extent necessary to meet the predetermined threshold. In order to determine the extent to which PA possesses the CCs, they must be asked to exercise them in the process of decision-making.

To test people’s capacity to acquire knowledge, CAs need to disclose information about the proposed course of action and ask a series of follow-up questions about: the nature of the procedure, its risks and benefits, the meaning of the terms they are using, and the methods they would use to check the reliability of information. The purpose of these checks is to ensure that PA isn’t merely repeating information they don’t understand and that they have the capacity to check and question the information they receive.

To establish that a person possesses the capacities to reason instrumentally and form and revise a life plan, CAs need to ask PAs to explain how they arrived at their decision to consume a particular pharmaceutical and what they intend to achieve by doing so (i.e., their goal). To test the validity of people’s inferences, CAs should ask PAs a series of follow-up questions aimed at: ascertaining whether they are choosing appropriate means to their goals, establishing why they believe the goal itself is valuable, and asking them to consider alternatives and explain why they are less desirable.

Filling in the details of Flanigan’s proposal to test competence before dispensing dangerous drugs reveals a number of safe-guards aimed at preventing people harming themselves as a result of their own incompetence. Firstly, individuals who fail competence assessments will be precluded from accessing drugs until they are competent, thereby protecting them from incompetently harming themselves. Secondly, the process of assessing and demonstrating competence forces people who are competent to offer reasons for their choices, answer questions about their values and thought processes, and show that they understand information disclosed to them. In short, competence assessments force people to think about their decision in the company of a knowledgeable bystander, providing them with opportunities to inform themselves and re-consider their intended course of action.

In this section, I have extended the proposal for reform outlined in *Pharmaceutical Freedom* to help allay two concerns opponents of drug liberalisation may have: (i) people unknowingly harming themselves, and (ii) incompetent people accessing dangerous drugs.

Firstly, I argued that in order to consider people to have knowingly consented to taking a drug, information about the risks must not only be available, it must also be accessible at the time the person makes the choice. Unless people have had the opportunity to acquire information by having it made accessible to them in a format they can understand, people cannot be said to have knowingly accepted the risk and given morally transformative consent. In order to ensure that people don’t unknowingly harm themselves, Flanigan’s proposal for liberalisation must be extended to include mandatory disclosures by pharmacists; bringing their obligations to disclose in line with those of clinicians seeking informed consent for treatment (Faden et al. 1986; Beauchamp and Childress 2008).

Secondly, I have extended Flanigan’s proposal by providing a brief account of what competence is and how it is assessed to illustrate how placing drugs behind the counter would safeguard incompetent people from harm. Before being sold dangerous pharmaceuticals, patients should be informed of the risks of the medication and should be asked follow up questions to ascertain whether they possess the capacities to acquire knowledge, reason instrumentally, and form and revise a life plan to the extent necessary to meet a competence threshold.

Having fleshed out the proposal, it is now time to see how it could work in practice. The goal is to show how, when taken together, the safe-guards outlined above could help people exercise their self-medication rights in pursuit of their best interests; protecting people from harms arising from their lack of knowledge or decision-making competence. To do this, in "Robertsville" I discuss a case of self-medication at greater length to illustrate how interactions between people and their pharmacists could work in a liberalised market for drugs. If drug liberalisation is ever to succeed in a democratic society, it will need extensive public support. To generate this support, we must try and allay the concerns of opponents of drug liberalisation. What is needed is a picture which has sufficient detail for us to see whether the functioning of the safe-guards outlined above yields intuitively plausible results. Providing this picture is the task of the next section.

## Robertsville

Robertsville is a small town. Like many small towns, it is famous for one thing only. In Robertsville’s case, it is for having recently granted citizens rights to self-medicate. Although Robertsville doesn’t have many amenities, it does have a doctors surgery and a pharmacist.

In Robertsville, people are now able to purchase recreational drugs, untested pharmaceuticals, and there are no longer prescription requirements. Although pharmacists are free to dispense them without a prescription, this doesn’t mean everyone can get hold of them. Pharmacists must assess people’s competence before dispensing drugs and engage in mandatory disclosures about the risks and benefits of consuming them, asking people to answer questions about the reasons for their decisions and whether they understand the information disclosed to them. People who cannot demonstrate they are competent at the time of purchase will not be dispensed medications.

The aim of all of these policies in Robertsville is to ensure that people don’t harm themselves unknowingly or as a result of their incompetence. To illustrate how they work in conjunction, let us consider an example:

Bruxism: For the last couple of weeks, Frank has been having terrible headaches when he wakes up in the morning. When he woke up today, he found a piece of his tooth-filling in his mouth and his face felt tense. Worried, Franks goes online and starts searching the NHS website for advice on whether to see a doctor. Online Frank discovers a condition called bruxism he had never heard of. The main symptoms are teeth grinding, which in turn leads to broken fillings and headaches. Frank also discovers bruxism is related to stress and can be a coping mechanism for frustration. Frank often feels both of these things, as he has a terrible boss. Convinced he has bruxism, Frank looks at how to treat it. One way to stop his headaches is to try and reduce his stress. Although Frank would like to do this, he has failed in the past because his fluctuating shift patterns at work make it difficult to engage with cognitive behavioural therapy. Another way to solve it is to adopt better sleep hygiene, which he can’t do because of his shifts. The third option is to give up on alcohol and recreational drugs such as MDMA or cocaine. Although Frank has cut down on these since his raving days, the occasional night out in a club is the only thing left in his life that still makes him feel young. Frank concludes that, given his inflexibility, there isn’t much use visiting the GP, who he presumes will encourage him to treat his underlying stress and lead a generally healthier life-style. Convinced there must be a way to reduce the symptoms he is feeling without dealing with the underlying issue, Frank embarks on more research. On the NHS website, Frank discovers that GPs occasionally prescribe muscle relaxants to treat bruxism. Having used Valium recreationally as a teenager, Frank feels it would help relieve his tension and decides to visit his pharmacists to get some Valium.

In *Bruxism*, Frank runs a high risk of misdiagnosing himself and, consequently, of choosing ineffective means to his ends; in this case, the absence of pain. Headaches are very common complaints and, in the majority of cases, aren’t caused by anxious teeth grinding. Some of the other causes of Frank’s headaches might be dehydration, eyesight problems, or not eating regular meals. If these are the true underlying causes, and they are left untreated by using Valium as a muscle relaxant, Franks headaches will likely continue. More worryingly Frank could find himself ignoring more serious problems which cause headaches (such as inflamed arteries in the head). Moreover, as Valium has psychoactive effects, using it to mask headaches could put Frank in a position in which he can’t identify further symptoms (such as drowsiness) that would be indicative of another explanation for his headaches.

In short, due to the potential for misdiagnosis and choice of inadequate treatment, Frank could be unknowingly harming himself by deciding to consume Valium to treat his headache. The question is: could the safe-guards set out in section "Some Unanswered Questions" help Frank avoid harming himself through ignorance or incompetence. To illustrate, it will be useful to expand on the example considered above:

Misdiagnosis: Frank has just made it to the pharmacists on Robertsville High Street. Frank goes inside and is greeted by the pharmacist, Mr. Nice. “What can I do for you?” asks Mr. Nice. Frank explains he would like to purchase some Valium. As these medicines are behind the counter, Mr. Nice tells Frank they will have to conduct a brief competence assessment. To start off, Mr. Nice asks Frank whether he is suffering from anxiety or depression, or whether this is for recreational use. “Neither,” Frank says, “I’ve had a terrible headache every morning for weeks and I think I’ve been grinding my teeth”. Frank tells Mr. Nice he thinks he has bruxism. Mr. Nice asks Frank whether a physician diagnosed him or whether he’d come to this conclusion himself, to which Franks responds he read it on the NHS website. “You should probably go to the doctor as your headaches seem persistent” says Mr. Nice, “but it is unlikely that this is bruxism so the Valium probably won’t help. Moreover, given that it makes you sleepy and makes your breathing shallow the best thing to do for your headache is to just go home, drink water and take some paracetamol.” Frank explains he hasn’t got the time to go home and convalesce, as he needs his headaches gone so he can go back to work and carry on with his life. Mr. Nice tells Frank he might find that harder on Valium than he currently does as it will make him sleepy and lethargic. Frank is worried about this possibility, but over the years of working shifts he has become quite adept at working even though he is tired. If it gets too bad, he will stop taking them.

Frank explains he doesn’t like going to the doctor and will look into potential other causes of his headache if it doesn’t go away after 2 or 3 weeks on Valium. He understands that there is a risk of misdiagnosis, but there isn’t really any way of completely excluding that risk either. When medical problems are complex, it can take more than one visit to a physician to get an accurate diagnosis. Getting an appointment at the GP is difficult, so he will try fixing it himself first and go to them if it doesn’t improve.

Mr. Nice tells Frank he shouldn’t drink alcohol when taking Valium, as they are both depressants. If taken together, they can lead to shortness of breath and, in worst case scenarios, death. Frank has always drunk every day, not excessively, but he does drink double or sometimes triple the UK national guidelines. Mr. Nice tells Frank it is unlikely he is going to stop drinking but that he is worried about the combined effect of the alcohol and Valium. Although he won’t go tee total, he will try and moderate his drinking.

Following this informational disclosure, Mr. Nice needs to assess the extent to which Frank is competent. The first thing to do is establish the competence threshold Frank must meet. During their conversation, Frank expressed reservations when confronted with the possibility of misdiagnosis, was clearly worried about the risk of combining Valium and alcohol, and was concerned about Valium interfering with his ability to work by making him sleepy and lethargic. Given that Frank is unlikely to stop drinking, change his mind about visiting the doctor, or trying less risky treatments (like resting and rehydrating, treating his underlying stress or simply wearing a mouthguard to prevent teeth-grinding), Mr. Nice concludes it is risky for Frank to consume Valium and sets a moderately high threshold of competence.^4^

Having agreed on the threshold, Mr. Nice needs to ascertain whether Frank can meet it. To do this, Mr. Nice needs to test the extent to which Frank possesses the Core Capacities (i.e., knowledge, rationality, and a life plan). To check whether Frank can acquire knowledge, Mr. Nice asks him to explain what bruxism is and asks him questions about the effects he thinks Valium will have, the outcome he intends to achieve by using Valium, and the risks he is taking by doing so. Frank responds that bruxism is teeth grinding, that this can lead to headaches, and that muscle relaxants are sometimes used to treat it. For Frank, the goal is to relax his jaw, thus lessening the headaches. Although he understands that there may be other causes for his headache, Frank insists that he wants to try Valium first. If that doesn’t work he will try something else or consult a doctor to see what they recommend. Frank is aware of the risks of misdiagnosis and has set a 2-3 week deadline on his attempts to self-medicate with Valium as a way of reducing the risk of him harming himself.

To test whether Frank possesses the capacity for instrumental rationality and the capacity to revise a life plan, Mr. Nice asks Frank to explain why he thinks it is so important to get back to work and why he can’t simply take some time off to recover. Mr. Nice tells Frank that his goal of getting on with his life and getting back to work may actually be hindered by using Valium to self-medicate, as it will make him sleepier. Getting some rest and trying to lead a generally healthier life, on the other hand, might actually help him further his goals more reliably for longer. Frank acknowledges that, in the long run, leading a healthier life would help him pursue his goals more effectively and that getting hooked on Valium would make it harder to get on with life. The problem is that leading a healthier life would require a lot of will power. Frank is moderately happy with how he lives his life. At the moment, it is just particularly stressful and Frank needs a short term solution which is easy to fit in to his routine. Valium seems, to Frank, to be the obvious answer.

Following their conversation, Mr. Nice determines that Frank is competent and dispenses Frank’s medications, informs him about recommended dosages and points out the number for a self-medication help-line printed on the packaging. “The helpline is staffed 24/7 by qualified professionals who can offer advice on how to use pharmaceuticals safely.” says Mr. Nice. “Call them if you have any questions or, alternatively, pop back in to the pharmacy.” With his medications in hand, Frank leaves the pharmacist.

The question we need to answer in *Misdiagnosis* is: if Frank came to harm from taking these medications, could this be attributed to his ignorance or incompetence? It seems to me that, if Frank does eventually come to harm from his Valium use, this cannot be said to have done so unknowingly or due to a lack of competence.

Prior to dispensing the medication, Mr. Nice informed Frank of the risks of consuming drugs and that of the existence of less harmful solutions to his headaches (e.g., rest, relaxation and hydration). Importantly, this information was disclosed to Frank at the time he was making the decision in a format he could understand. In other words, the information was made accessible to him (as opposed to simply being made available). Moreover, the fact that competence assessments require Frank’s active participation serves to guard against the possibility of Frank simply sitting through the disclosure, letting it go in one ear and out the other. Given that Frank must demonstrate that he has the capacity to acquire knowledge before Mr. Nice can dispense the medication, Frank has to engage with the informational disclosure and take it into account in his deliberations.

If, for example, Frank didn’t understand that he could be mistaken about the cause of his headaches and that, as a consequence, the Valium might not help, he would have been found to be incompetent by Mr. Nice and Mr. Nice would not have dispensed the medications Frank had asked for. This would also be the case if Frank’s lack of competence were down to the fact that Frank couldn’t demonstrate he possessed the capacity for instrumental rationality when choosing treatment options, or if Franks behaviour was completely purposeless in that he didn’t have a goal he was seeking to achieve by taking Valium.

In short, informational disclosure and competence assessments could help protect people from unknowingly or incompetently harming themselves without abridging people’s self-medication rights. If extensive safe-guards are in place to ensure that people purchasing drugs are both competent and have the information about risks when they need it, the idea that people should be entitled to access dangerous pharmaceuticals without a prescription is less counter-intuitive. Under a regime of liberalised access, such as the hypothetical one I propose here, individuals who wish to avoid harming themselves have ample opportunities to engage with experts who can disclose information to them and answer any questions they may have to help them understand the risks they are taking. Once information has been made accessible to people and their decision-making competence has been rigorously assessed, any harm that may result from their actions cannot be attributed to ignorance or incompetence.

It could be objected at this point, that the hypothetical scenario I have outlined above is overly optimistic in a couple of ways. Firstly, it might objected that it is unlikely, in practice, that pharmacists will take the time to have conversations about the drugs and ascertain competence. Isn’t it more likely they will simply dispense the medication, no questions asked? Secondly, it could be objected that I haven’t provided any data to justify the optimistic view of how things would progress in Robertsville and, as a consequence, it is still unclear whether the safeguards outlined above would help prevent people from harming themselves with drugs. In what remains of this section, I take up these objections.

Let us take the first objection first. Assessing competence and disclosing information in an accessible format is time-consuming. Implementing the extensions to Flanigan’s proposal I am arguing for will, therefore, mean that dispensing medications in Robertsville is likely to be a more protracted process than simply having a prescription filled. Now, the problem is that time is in short supply in many healthcare systems,^5^ which could make compliance difficult to achieve (Hibbert et al. 2002, p. 55, Berger 2009). Community pharmacists in particular, are under commercial pressure to fill prescriptions quickly to increase the amount of patients they can see (Berger 2009; Latif 2000; Wingfield et al. 2004; Hibbert et al. 2002; Prayle and Brazier 1998; Resnik et al. 2000). The question is then: how likely would it be in practice that pharmacists would engage in thorough competence assessments and information disclosure before dispensing drugs?

The answer to this question will depend on a number of factors including (among others): whether dispensers have the resources to meet these requirements, whether having these conversations is a legal requirement (Resnik et al. 2000), what the sanctions for non-compliance are, how effectively these are enforced, how well the skills are taught (Coulter and Ellins 2006, p. 68), how much emphasis is placed on students learning them during training (Roche and Kelliher 2009; Kettle 2003; Wingfield et al. 2004), the institutional culture of the workplace (Latif 2000; Resnik et al. 2000), and whether there is a widespread expectation amongst the public that pharmacists routinely disclose information about drugs and assess competence. In short, how likely compliance will be will depend on the specifics of how the proposal outlined above is implemented on the ground.

The question of how to implement policies to achieve optimal compliance is beyond the scope of this paper. The reader is therefore invited to introduce their preferred view of how to enforce the proposal outlined above. As a consequence, I’m forced to leave the question of how likely compliance with my proposal will be in practice unanswered in this paper. That said, what this short discussion of enforceability does reveal, however, is that how likely compliance will be with any given proposal is not a fixed, immutable, characteristic. Instead policymakers have a number of tools at their disposal which they can use to increase the likelihood of compliance.

The requirements that pharmacists assess competence and disclose information before dispensing drugs mirrors informed consent requirements imposed on other front-line healthcare professionals. Seen in this light, achieving compliance with the policies outlined in this paper doesn’t seem especially problematic. If backed by appropriate enforcement mechanisms (whatever those turn out to be), lack of compliance with the policy can be made as likely as compliance with informed consent requirements in other contexts. Although there are still hurdles to be overcome when implementing informed consent requirements consistently in practice (Coulter and Ellins 2006; Evans et al 2007; Jackson and Warner 2002; Kim 2010, p. 59), these are not generally considered insurmountable or a reason to cease trying to implement the requirements of informed consent. Instead, they are seen as problems to be overcome by, for example, redirecting resources or developing new and betters ways of ensuring compliance. This is also the approach we should take to the problem of ensuring pharmacists comply with their obligations to assess competence and inform people before dispensing drugs.

Having responded to the first objection, it is now time to consider the second objection: the fact that I have not provided data to support my account of how things would progress in Robertsville. The crux of this objection is that, without data on how effective the policies outlined above are at protecting people from harm, we can’t be sure that the deontological and the epistemilogical-consequentialist arguments will pull in broadly the same direction. This is a problem for defenders of drug liberalisation, because unless we can allay concerns that increased access to drugs will lead to catastrophic consequences for significant numbers of people, it will be hard to generate the levels of public support for drug liberalisation necessary to implement the policy in a democratic society.

The short response to why I haven’t provided any data on how effective the proposals outlined above would be at ensuring that people don’t harm themselves as a result of ignorance of their own incompetence is that we simply don’t have it. The reason we don’t have any directly relevant data is that, to my knowledge, the proposals I am outlining haven’t been implemented anywhere in the world. Given the lack of direct data, if we are to try and allay concerns about the liberalisation of drugs, we need to search for analogous evidence that might give us an indication of whether the proposals outlined are workable in practice. However, once we choose to go down this route we are immediately confronted with the problem of determining what counts as "analogous evidence", which turns out to be far from easy.

To illustrate, one option would be to try and compare the drug poisoning rates in countries with stringently enforced prescription requirements with the rates in countries with more lax approaches to prescriptions (Peltzman 1987). The idea behind making these comparisons is that people in countries with lax enforcement of prescription requirements are living in a de facto (but not *de jure*) liberalised market, which is the closest we can get to a certification scheme. If there were significantly higher drug poisoning rates in countries with de facto liberalised markets, we could take this as an indication that liberalising access to pharmaceuticals could have severe negative consequences.

The problem with this approach is that, although data is available for some countries accurate data on drug poisonings rates in countries which do not rigorously enforce prescription requirements is hard to come by, making it impossible to establish fair comparisons between the two groups. Furthermore, even if the data concerning mortality and morbidity rates did consistently exist and showed that both were higher in countries which don’t enforce prescription requirements, it is not clear that alone would show the harm reduction measures I am arguing for are ineffective. In order for the data to establish that, we would need good evidence that pharmacists dispensing medications actually abide by the harm reductions outlined above. In the absence of said evidence, higher mortality and morbidity rates don’t show us that disclosure and competence assessments are insufficient to protect people from harm.

A second option is to look for data on how the decriminalisation of drugs affects consumption, overdose, and death rates. The idea is that the decriminalisation of recreational drugs is partly analogous to a certification system because both proposals make it easier to access drugs. If decriminalisation has disastrous consequences, we might argue, this is a reason to be cautious of all proposals aimed at liberalising access to drugs.

According to Flanigan, “When Portugal de-criminalised all recreational drugs in 2000, rates of abuse, overdose, and HIV infection fell, and greater numbers of users sought rehabilitation and treatment” (Flanigan 2017, p. 80). Data from Czechia also points to decreased use after the decriminalisation of recreational drugs in 2009 and drug death rates which are below the EU average (EMCDDA 2019). We might think, therefore, that the experience of decriminalisation provides evidence to allay the concerns opponents of drug liberalisation might have to implementing a certification scheme.

The problem with these comparisons stems from the fact that that decriminalisation is only partly analogous to the regulatory regime I am advocating. Firstly, decriminalisation only applies to recreational drugs. As a consequence, the data doesn’t give us a clear indication of what to expect from abolishing prescription requirements across the board. Secondly, with decriminalisation, trade in recreational substances still takes place in an unregulated marketplace where, for all intents and purposes, it is impossible to enforce the requirement that dealers establish competence and disclose information about the drugs they distribute. As a consequence, it is unclear what data concerning prevalence of use and overdose rates under a decriminalised system means for the viability of the proposals outlined above.

A third option would be to look at the effects disclosure and competence assessments have in other healthcare settings, such as clinical consultations between physicians and patients. The data here is mixed. There is some evidence to suggest that better communication practices during the process of disclosure can lead to better medication adherence and improved patient knowledge, but have mixed results on health outcomes (Coulter and Ellins 2006, p. 71; Adams 2010, p. 66) and the prevention of adverse events (Coulter and Ellins 2006, p. 143). With regard to the effectiveness of competence assessments, there is some evidence to suggest that higher levels of decision-making competence correlate with the avoidance of negative outcomes (Parker et al. 2015; Bruine de Bruin et al. 2007), which could be seen to lend support to the idea that properly conducted competence assessments could reduce adverse drug events, such as accidental poisonings.

Given the mixed results, it is difficult to assess how effective mandatory disclosure and competence assessments would be at reducing harms resulting from either ignorance or incompetence. Here, as before, we also face the problem of applying the insights from data gathered during clinical interactions in doctors offices and hospitals to interactions in the pharmacy, which are not necessarily comparable.

To summarise, due to the fact that the regulatory regime outlined above hasn’t been implemented in practice, we can’t be sure how effective the proposals outlined above will be at ensuring that people don’t harm themselves as a result of their own ignorance or incompetence. In other words, there is an inherent uncertainty as to what the effects of liberalising access to drugs would be in practice. As a consequence of this lack of empirical evidence, it will prove difficult to allay the concerns of the staunchest opponents of liberalising access to drugs. The problem is that, unless the proposal is actually implemented, it will be impossible to ever generate the kind of robust evidence that would convince those most opposed to drug liberalisation that the deontological and consequentialist arguments can be made to pull in broadly the same direction.

Flanigan’s solution to this Catch-22 situation is to argue that self-medication rights ought to be insulated from democratic politics. Rights to access drugs, like other important rights, should be enforced by the judiciary (Flanigan 2017, p. 163). My reservation with this solution is that, although using the judiciary to enforce self-medication rights is a means of side-stepping the (potentially biased) opposition to liberalisation, it does nothing to resolve the genuine uncertainty surrounding the effects of liberalising access to drugs or actually allay concerns surrounding liberalisation.

If we genuinely want to allay the concerns of opponents of drug liberalisation, we have to actually resolve the uncertainty surrounding the effects of moving to a certification scheme. Judicial decisions won’t help here. What we need is high quality data on the effects of removing prescription requirements. Until we have this data, we cannot be sure that protecting people’s right to self-medicate won’t have negative consequences for society (Dunn 1997). Under these conditions of uncertainty, opponents of drug liberalisation have genuine reasons to be concerned which must be taken seriously.

As I see it, there are two ways of getting the data we need to ascertain whether or not the deontological and consequentialist arguments can be made to pull in broadly the same direction. The first is to wait for a natural experiment to occur and use the results to extrapolate to other situations. The second is to conduct a policy trial consisting in implementing a certification scheme in a limited geographical area for a limited period of time for the purposes of tracking key measures such as overdose deaths, prevalence of use of drugs, health outcomes, what healthcare services are being used and healthcare cost. This data could also be supplemented with qualitative data gathered through interviews of surveys of a subset of the population.

My preference would be for the latter. There are two reasons for this. Firstly, the results of natural experiments can be difficult to interpret and, as a consequence, it might be harder to establish precisely what the experiment has shown. Conducting a policy experiment allows us more control over the regulatory regime being tested and, therefore, helps ensure that the results are applicable to the problem at hand (McDermott 2002, p. 39). Secondly, there is no guarantee that a natural experiment will ever occur and, if it does, we might find ourselves waiting for a long time. Given how high the stakes are when it comes to drug regulation, we need data sooner rather than later.

## Conclusion

In this paper, my aim has been to try and allay what I believe to be one of the main worries surrounding drug liberalisation: that doing so will lead to people unknowingly or incompetently harming themselves. I have argued that appropriate safeguards could help make granting people self-medication rights compatible with preventing them from harming themselves as a result of ignorance or lack of decision-making capacity. In "Pharmaceutical Freedom", I outlined Flanigan’s view and argued that, as it stands, it lacks the detail necessary to see how it would apply it in practice. As a consequence, it is difficult to see whether the safe-guards it incorporates could be sufficient to prevent people from harming themselves. In other words, granting everybody self-medication rights might lead to some people thwarting their best interests instead of advancing them. This is worrying for defenders of liberalised access to drugs because the case for self-medication rights is strongest when the epistemological-consequentialist and deontological arguments pull in the same direction. If granting people the right to make intimate bodily choices leads to people reliably acting against their best interests, it is unlikely that the policy will be successful in a democratic society.

To help ensure that the two arguments pull in broadly the same direction, in "Some Unanswered Questions" I proposed extending Flanigan’s proposal in two ways. First, I argued that in order to help prevent people from unknowingly harming themselves by using drugs, information needs to be made accessible to them at the time of purchase in a format they can understand. In other words, under the proposed hypothetical regulatory regime, pharmacists would need to have an expanded duty to disclose information about drugs to those who wish to purchase. Extending Flanigan’s account in this way brings disclosure requirements for self-medication rights in line with standard views of the doctrine of informed consent, which normally require that clinicians disclose information and check understanding before considering someone’s acquiescence to treatment a form of morally binding consent. Second, I briefly provided an account of what competence is and how it ought to be assessed to fill in the details of Flanigan’s proposal that dangerous drugs should be placed behind the counter to enable pharmacists to screen for capacity.

Finally, in "Robertsville", I used an extended thought experiment to provide a picture of what a hypothetical liberalised market for drugs could look like and illustrate how these safe-guards could work together to help prevent people from unknowingly or incompetently harming themselves in a liberalised market for drugs. I have argued that, although disclosure requirements and competence assessments won’t stop all people harming themselves by using drugs, checking competence, and making information accessible to people at the time they need it could help ensure that people understand the risks they are taking and would provide them with opportunities to reconsider their decision. Individuals who choose to take these risks may come to harm but, importantly, this cannot be attributed to ignorance or lack of decision-making competence.

In conclusion, if suitably extended, Flanigan’s proposal for drug liberalisation could strike a plausible balance between allowing competent people to access drugs for the purposes of self-medication (or recreation) and protecting people from harms caused by their own ignorance or incompetence. Whether Flanigan’s proposal will work in practice will depend on a number of factors such as whether pharmacies have sufficient resources to meet the requirements, whether institutional cultures support compliance with ethical demands, what the sanctions for non-compliance are, and how well these are enforced. Although ensuring compliance with policies aimed at reducing harms resulting from ignorance or incompetence won’t always be straightforward, these problems are not unassailable. In this sense, the hypothetical regulatory regime proposed in this paper is no different to any other informed consent requirements.

For the most fervent opponents of drug liberalisation, the hypothetical case presented above will be insufficient to convince them that liberalised access to drugs is compatible with preventing people from harming themselves through ignorance or incompetence. For them, only hard data will do. In a democratic society, these concerns deserve to be taken seriously. The case for liberalisation is, therefore, still incomplete. What remains to be done is test proposals for liberalisation in practice. What I hope the hypothetical regulatory regime outlined above *has* shown is that liberalisation is worth testing.

## Footnotes

1 For a discussion of the potential impact of self-medication rights on the doctor-patient relationship see: Flanigan (2017, p. 218).

2 Some accounts of competence also include the requirement that people be able to appreciate the consequences of their decisions (Appelbaum 2007). Appreciation "refers to a patient’s recognition that information given to them about their disorder and potential treatment is *significant for* and *applicable to* their own circumstances" (Grisso et al. 1995, p. 128). My preferred interpretation of the appreciation criterion is that appreciation is an element of correct understanding and not a category in and of itself. Readers who disagree are invited to adopt their preferred account of competence.

3 On non-risk sensitive accounts of competence, the threshold of competence people have to meet can be set independently of the risk or of the person’s values. On these accounts, it may not be necessary to have a two-way conversation to establish the threshold. What is inescapable, however, is the need to adopt some procedure for setting the threshold.

4 On non-risk sensitive accounts of competence, this first step is not necessary as the height of the threshold isn’t dependent on risk. As a consequence, it can be established without consulting the person’s values.

5 Although robust data on how long pharmacists spend with customers on average before dispensing medication is hard to come by, there are some estimates in the literature. David Resnick and colleagues estimate that, in the USA, pharmacists working in busy pharmacies fill up to 160 prescriptions a shift, or one every 3 to 6 min (Resnik et al. 2000). Bruce Berger reports a similar estimate of 2–5 min (Berger 2009).
